# Unraveling the central and bridge psychological symptoms of people living with HIV: A network analysis

**DOI:** 10.3389/fpubh.2022.1024436

**Published:** 2023-01-04

**Authors:** Huan Wen, Zheng Zhu, Tiantian Hu, Cheng Li, Tao Jiang, Ling Li, Lin Zhang, Yanfen Fu, Shuyu Han, Bei Wu, Yan Hu

**Affiliations:** ^1^School of Public Health, Fudan University, Shanghai, China; ^2^School of Nursing, Fudan University, Shanghai, China; ^3^Fudan University Centre for Evidence-based Nursing: A Joanna Briggs Institute Centre of Excellence, Fudan University, Shanghai, China; ^4^Shanghai Public Health Clinical Center, Fudan University, Shanghai, China; ^5^School of Nursing, Dali University, Dali, Yunnan, China; ^6^School of Nursing, Peking University, Beijing, China; ^7^NYU Rory Meyers College of Nursing, New York University, New York City, NY, United States

**Keywords:** HIV/AIDS, PLWH, psychological network, network structure, symptom management

## Abstract

**Background:**

People living with HIV (PLWH) experience multiple psychological symptoms. Few studies have provided information on central and bridge psychological symptoms among PLWH. This information has implications for improving the efficiency and efficacy of psychological interventions. Our study aimed to identify the central and bridge psychological symptoms of PLWH and to explore the interconnectedness among symptoms and clusters.

**Methods:**

Our study used data from the HIV-related Symptoms Monitoring Survey, a multisite, cross-sectional study conducted during 2017–2021. We used *R* to visualize the network of 16 symptoms and analyzed the centrality and predictability indices of the network. We further analyzed the bridge symptoms among the three symptom clusters.

**Results:**

A total of 3,985 participants were included in the analysis. The results suggested that sadness had the highest strength (*r*_*S*_ = 9.69) and predictability (70.7%) compared to other symptoms. Based on the values of bridge strength, feeling unsafe (*r*_*bs*_ = 0.94), uncontrollable worry (*r*_*bs*_ = 0.82), and self-abasement (*r*_*bs*_ = 0.81) were identified as bridge symptoms. We also found a strong correlation between sadness and self-abasement (*r* = 0.753) and self-loathing and self-blame (*r* = 0.744).

**Conclusion:**

We found that sadness was the central psychological symptom of PLWH, indicating that sadness was the center of the psychological symptom network from a mechanistic perspective and could be a target for intervention. Deactivating bridge symptoms, including “feeling unsafe,” “self-abasement,” and “uncontrollable worry,” could be more effective in preventing symptom activation from spreading (e.g., one symptom activating another).

## Introduction

According to the Joint United Nations Program on HIV/AIDS (UNAIDS), as of 2021, there were 38.4 million people living with HIV/AIDS (PLWH) worldwide (1). PLWH commonly experience multiple psychological symptoms due to the diagnosis of the disease, stigma, social discrimination and the side effects of antiretroviral therapy (2). Previous studies have shown that the prevalence of psychological symptoms is significantly higher in PLWH than in the general population (3, 4). A recent study in China indicated that 61% of PLWH had depressive symptoms (5). Kendall et al. (6) conducted a cross-sectional study and found that 41% of PLWH reported multiple simultaneous psychological symptoms compared to 22% of adults without HIV. High symptom prevalence and severity not only contribute to great mental suffering but also lead to physical comorbidities and a lower quality of life (7, 8). Another study focused on the psychological symptoms of PLWH confirmed that psychological symptoms play a role in risky sexual behaviors and that interventions targeting psychological symptoms might prevent the occurrence of risky sexual behaviors (7). Levy et al. (8) examined the associations between psychosocial risk factors and subclinical atherosclerosis among women living with HIV and among HIV-negative women and concluded that psychological symptoms could increase the risk of atherosclerosis in women living with HIV. PLWH living in poor areas also have a higher number of mental disorders or psychological symptoms because they tend to have less access to health care services (9, 10).

Current interventions for psychological symptoms in PLWH have some limitations; for example, they may ignore the interaction among multiple symptoms and strength of symptom connection experienced by PLWH in the real world. This may lead to low efficiency and accuracy in the management of co-occurring symptoms. For PLWH, the persistent range of psychological reactions is often caused by strong interactions and feedback loops among various negative emotions (11). By constructing a symptom network and finding symptoms with high centrality, we can identify the most influential symptoms in the network and discover the role of central symptoms on other symptoms, which would allow us to focus on precise symptom intervention (12). Bridge symptoms connect different clusters of symptoms or subgroups of symptoms within the same cluster (13). If we can identify the central and bridge symptoms among symptom clusters in PLWH, the effectiveness and efficiency of psychotherapeutic interventions could be increased.

### Central and bridge symptoms

Central symptoms are identified through the centrality indices of strength, betweenness, and closeness. Strength is an indicator of network connectivity and corresponds to the sum of the absolute edge weights connected to each node (14). Betweenness indicates the frequency of a node on the shortest path between other nodes. Closeness represents the inverse of the sum of distances from one node to all other nodes in the network (14). Among these three centrality indices, strength has been most emphasized. Some studies have concluded that betweenness centrality and closeness centrality are less applicable (15–17). Bringmann et al. (12) found that for psychological networks, betweenness and closeness were not suitable indices of node importance. We usually identify bridge symptoms based on bridge centrality indices (18).

Previous studies have shown that central and bridge symptoms play important roles in clinical psychotherapy for PLWH (19, 20). Central symptoms are the most central symptoms in the symptom network from a mechanistic perspective. They are strongly correlated with and have the most interactions with other symptoms in the symptom network. Therefore, targeting central symptoms for psychological intervention treatment can accelerate the deactivation of the network and improve the precision and efficiency of the intervention (19, 21–23). Although there is now controversy about bridge symptoms, most studies have shown that bridge symptoms are associated with comorbidity and cluster structures in the symptom network (13, 24–26). Comorbidity and clusters can impede the progress of interventions to treat psychological symptoms; therefore, bridge symptoms also deserve priority attention (27–29). Previous studies have suggested that interventions targeting bridge symptoms are more effective than those targeting other symptoms (18, 27–30).

However, few studies provided information regarding central and bridge symptoms in PLWH, which is crucial for enhancing the efficiency and efficacy of psychological interventions. Therefore, we aimed to identify the central and bridge psychological symptoms of PLWH and to explore the complex interconnectedness among symptoms by developing symptom networks.

## Methods

### Sample

Our study used data from the HIV-related Symptoms Monitoring Survey (HSMS), a multisite, cross-sectional dataset of Chinese PLWH collected by the authors during 2017–2021. Participants were recruited from 11 cities in eastern (Shanghai), central (Hengyang), and southwestern (Nanning, Ruili, Kunming, Tengchong, Longxing, Changning, Baoshan, Lincang, and Changning) China. The inclusion criteria were as follows: (1) diagnosed with HIV infection and (2) aged 18 years or older. The exclusion criteria were as follows: (1) unable to complete the self-rating scale and (2) a diagnosis of an HIV-associated neurocognitive disorder based on the DSM. A total of 3,985 questionnaires were obtained. All questionnaires were distributed and collected on the spot. Our study obtained the consent of the hospital. Written informed consent was obtained from all subjects before the study. The Institutional Review Board of Fudan University School of Nursing approved this study (IRB# TYSA2016-3-1, IRB# TYSQ2020-4-06). Additional details on the HSMS have been published elsewhere (31, 32). This questionnaire has been used in many published studies (32, 33).

### Measures

#### Sociodemographic and clinical data

We used a self-administered questionnaire to collect sociodemographic and clinical data. The sociodemographic variables included age, gender, ethnicity, education level, employment status, marital status, and primary caregiver. The clinical data included years since HIV diagnosis and latest CD4+ T-cell count.

#### Self-reported symptoms

We used a self-administered questionnaire to collect information on 16 highly prevalent HIV/AIDS-related psychological symptoms. These symptoms were selected based on previous studies and have high prevalence and high specificity for PLWH (32). In our team's previous study, according to severity, the 16 psychological symptoms were categorized into three clusters: emotions, personality traits, and cognitive processes (33). Emotions refer to a temporary state of mind or feeling. Symptoms in the emotion cluster can fluctuate and change over short periods, while symptoms in the personality trait cluster are persistent over time (34). Personality traits refer to a relatively stable, consistent, and enduring internal characteristic that is inferred from a pattern of behaviors, attitudes, feelings, and habits in the individual (34). Cognitive processes refer to the mental function involved in acquisition, storage, and interpretation (35). Emotions included “feeling nervous, anxious or on edge,” “feeling down, depressed or hopeless,” fear, anger, panic, sadness, loneliness, shame, and feeling overwhelming pressure. Personality traits included “not being able to stop or control worrying,” “little interest or pleasure in doing things,” impulsivity, and feeling unsafe. Cognitive processes included self-abasement, self-loathing, and self-blame (33). The participants were asked, “During the last 4 weeks, did you have the following symptoms? If you did, please rate the severity of these symptoms.” Symptoms were scored 0–3, with higher scores indicating more severe symptoms. To evaluate the psychometric properties of the questionnaire, we included five experts for validation. The questionnaire showed good validity and reliability (33). In our sample, the questionnaire showed high expert validity (the content validity index was 0.918) and internal consistency (Cronbach's α = 0.961) (33).

### Data analysis

#### Symptom networks

Statistical analyses were conducted using SAS 9.4. The demographic characteristics were described using frequencies, percentages, means, and standard deviations. We used the *qgraph* package in *R* to construct an undirected network model with all 16 symptoms. In the symptom network, each node represented one symptom, and the edges between two nodes represented Spearman correlation relationships between two symptoms. The thicker the edge was, the stronger the correlation between the two symptoms (18, 36–38). We used *r* to indicate the Spearman correlation coefficient. The Fruchterman-Reingold algorithm was used to visualize the network (39), with the strongest correlation nodes placed in the center of the network and nodes with similar characteristics placed relatively closer, while nodes with weak and few connections were placed on the periphery of the symptom network.

#### Centrality, bridge centrality and predictability

Strength centrality was used to identify central symptom. We used the *R* package *qgraph* to conduct centrality analysis. The strength is an indicator of network connectivity. When a symptom has a higher strength centrality, it is more likely to co-occur with other symptoms. The betweenness indicated the frequency of a node on the shortest path to any two other nodes. When a node had a higher betweenness centrality, it had more influence on the network. The closeness represented the average distance from a node to all other nodes. The larger the value of closeness centrality was, the shorter the overall path from that node to other nodes in the network was. Among these three centrality indices, strength was the main centrality indicator. Previous studies concluded that betweenness centrality and closeness centrality were less applicable (15, 40). Bridge strength centrality was used to identify bridge symptoms. Bridge symptoms were broadly defined as symptoms that connect different clusters of symptoms (13). The *R* package *networktools* was used to identify bridge symptoms and bridge strength among the three clusters. We used *r*_*S*_ to indicate the strength centrality index and *r*_*bS*_ to indicate the bridge strength centrality index.

We used the *R* package *mgm* to calculate the predictability for each node. Predictability is a metric that reflects the extent to which a node's variation can be explained by the variation in its connected nodes. The average predictability of all nodes in a network is usually used to reflect the extent to which the symptom network is influenced by external factors. When the average predictability of a group's symptom network is low, the difficulty of using external factors to alleviate symptoms may be higher (41). We used *r*_*p*_ to indicate the predictability.

#### Accuracy, stability and difference tests

Bootstrapping methods were performed to assess the accuracy and stability of the network by using the *R* package *bootnet*. The accuracy was evaluated by calculating the 95% confidence intervals (CIs) for the edge weight values. We used non-parametric bootstrapping (1,000 bootstrap samples) to construct the CIs (42). The stability was evaluated by calculating the correlation stability coefficient of the strength centrality using a case-dropping subset bootstrap (1,000 bootstrap samples) (15, 16). The correlation stability coefficient should preferably be >0.5 but at the very least >0.25 (43). Finally, to identify whether the estimations of network connections and centrality for different variables differ, we performed bootstrapped difference tests among edge-weights and centrality indices in the LASSO regularization of partial correlation networks based on polychoric correlation matrices (15, 16). We used *r*_*cS*_ to indicate the correlation stability coefficient. The significance was set at α = 0.05.

## Results

### Participant characteristics and prevalence and severity of symptoms

Table 1 shows the sample characteristics. The mean age of the participants was 39.60 ± 11.81 years, ranging from 18 to 87 years old. Among all participants, 73.5% were male. The majority of participants were employed (*n* = 3,818, 98.4%) and married (*n* = 1,705, 48.0%). Most of them were cared for by family members (*n* = 2,333, 58.9%). The majority had been diagnosed with HIV for 6 months to 3 years (*n*_2_ = 1,213, 30.6%), followed by 5–10 (*n*_4_ = 1,018, 25.7%), 3–5 (*n*_3_ = 620, 15.7%), >10 years (*n*_5_ = 571, 14.4%) and <6 months (*n*_1_ = 537, 13.6%). Most of them had undergone ART for <1 year (*n* = 1,055, 27.1%), followed by 5–10 (*n* = 984, 25.3%), 1–3 (*n* = 871, 22.4%), 3–5 (*n* = 648, 16.7%) and >10 years (*n* = 333, 8.6%).

**Table 1:** Description of sociodemographic and clinical characteristics of the sample (*N* = 3,985).

**Variable**	**Mean ±SD or *N* (%)**
Age, years	39.61 ± 11.85
Male	2,929 (73.50%)
**Race/ethnicity**	
Han	3,265 (81.93%)
Minority	720 (18.07%)
**Education level**	
Middle school or below	1,954 (49.03%)
High school or equivalent	867 (21.76%)
Postgrad or equivalent	1,035 (25.97%)
Master's or above	129 (3.24%)
**Employment status**	
Employed	3,923 (98.44%)
Otherwise	62 (1.56%)
**Marital status**	
Single	1,328 (33.32%)
Married	2,135 (53.58%)
Otherwise	522 (13.10%)
**Primary caregiver**	
Myself	1,508 (37.84%)
Family members (spouse, kids, or other relatives)	2,359 (59.20%)
Otherwise	118 (2.96%)
**Years since HIV diagnosis**	
< 6 months	537 (13.48%)
6 months−3 years	1,239 (31.09%)
3–5 years	620 (15.56%)
5–10 years	1,018 (25.55%)
>10 years	571 (14.33%)
**CD4**+**T-cell count**	
< 50	274 (6.88%)
50–350	1,211 (30.39%)
350–500	785 (19.70%)
>500	1,715 (43.04%)

Table 2 presents the prevalence and severity of all psychological symptoms. Feeling overwhelming pressure (*n* = 1,489, 37.37%) was the most prevalent symptom, followed by little interest or pleasure in doing things (*n* = 1,230, 30.87%) and feeling down, depressed or hopeless (*n* = 1,180, 29.61%). Feeling overwhelming pressure (0.55 ± 0.82) was the most severe symptom, followed by little interest or pleasure in doing things (0.40 ± 0.68) and feeling down, depressed or hopeless (0.39 ± 0.68).

**Table 2:** Severity and predictability of psychological symptoms.

**Symptoms**	**Abbreviation**	**Mean**	**SD**	**Prevalent**	**Predictability**
Feeling nervous, anxious or on edge	Feeling nervous	0.36	0.65	28.38%	0.70
Feeling down, depressed or hopeless	Feeling down	0.39	0.68	29.61%	0.65
Fear	Fear	0.28	0.62	20.58%	0.68
Anger	Anger	0.24	0.58	17.79%	0.58
Panic	Panic	0.25	0.58	19.42%	0.68
Sadness	Sadness	0.30	0.62	22.53%	0.71
Loneliness	Loneliness	0.36	0.69	25.87%	0.57
Shame	Shame	0.27	0.64	19.07%	0.56
Feeling overwhelming pressure	Feeling overwhelming pressure	0.55	0.82	37.37%	0.54
Not being able to stop or control worrying	Uncontrollable worry	0.31	0.61	23.69%	0.67
Little interest or pleasure in doing things	Little interest	0.40	0.68	30.87%	0.52
Impulsivity	Impulsivity	0.24	0.56	18.17%	0.56
Feeling unsafe	Feeling unsafe	0.30	0.65	20.20%	0.60
Self-abasement	Self-abasement	0.32	0.65	24.07%	0.66
Self-loathing	Self-loathing	0.22	0.57	16.26%	0.68
Self-blame	Self-blame	0.30	0.66	20.73%	0.67

We conducted regression analyses of age, gender, education level, employment status, marital status, primary caregiver, years since HIV diagnosis, and CD4+T-cell count with the total score of symptoms, and finally identified the covariates as age (*P* < 0.05), marital status (*P* < 0.05), primary caregiver (*P* < 0.05), and years since HIV diagnosis (*P* < 0.05). Covariates were controlled for in all network analyses.

### Symptom networks

**Figure 1:**
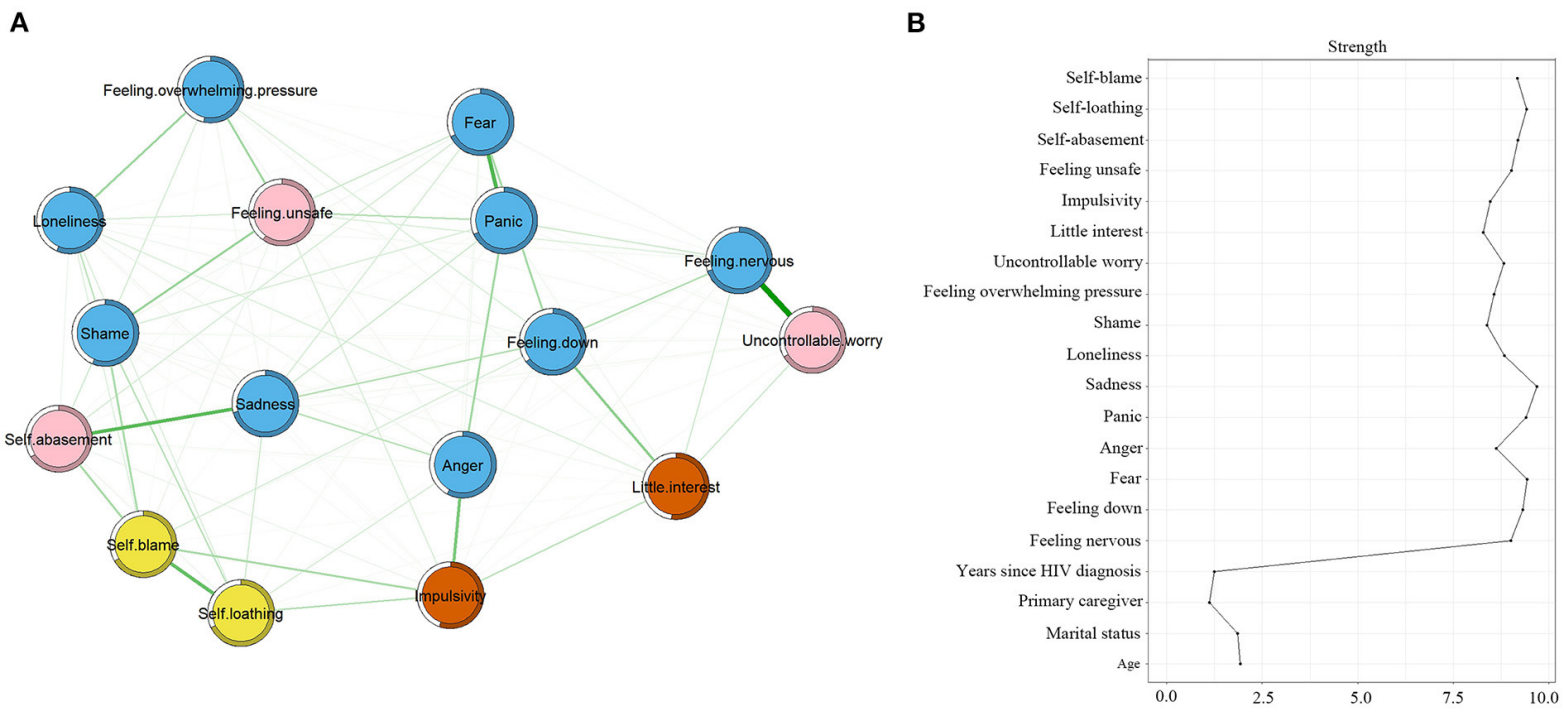
Symptom network and centrality index. **(A)** Symptoms network; **(B)** strength centrality index of the network. The same color represents the same symptom clusters and pink represents bridge symptoms. Bridge symptoms: feeling unsafe, self-abasement, and uncontrollable worry; Emotions: feeling nervous, feeling down, fear, anger, panic, sadness, loneliness, shame, feeling overwhelming pressure; Personality traits: uncontrollable worry, little interest, impulsivity, feeling unsafe; Cognitive processes: self-abasement, self-loathing, self-blame.

Figure 1A shows the network of psychological symptoms. All the edges were positive. We found that four edges, uncontrollable worry and feeling nervous (*r* = 0.79), fear and panic (*r* = 0.75), sadness and self-abasement (*r* = 0.75), and self-loathing and self-blame (*r* = 0.74), were thicker than other edges, which means that these relationships were stronger than other correlations (*P* < 0.05).

### Centrality, bridge centrality and predictability

**Figure 2:**
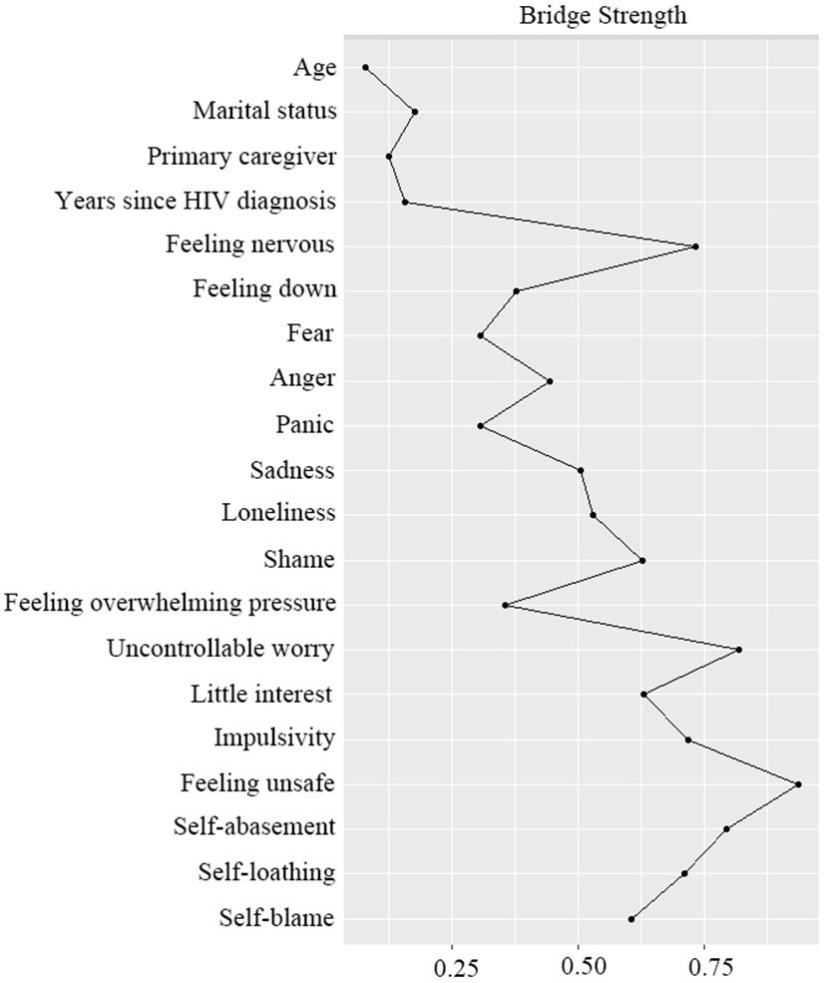
Bridge strength centrality index of the network.

Figure 1A shows the bridge symptoms of the three domains. Figure 2 shows that feeling unsafe had the highest bridge strength centrality (*r*_*bS*_ = 0.94), followed by uncontrollable worry (*r*_*bS*_ = 0.82) and self-abasement (*r*_*bS*_ = 0.81). The pink nodes represent bridge symptoms. Based on the values of strength centrality, feeling unsafe, self-abasement, and uncontrollable worry were identified as bridge symptoms. “Uncontrollable worry” and “feeling unsafe” connected the personality traits and emotion clusters, and “self-abasement” connected the cognitive processes and emotion clusters.

Figure 1B presents the strength centrality index. Sadness had the largest values for strength (*r*_*S*_ = 9.69), followed by fear (*r*_*S*_ = 9.44) and self-loathing (*r*_*S*_ = 9.41). Predictability is presented as a circle around the node in Figure 1A. Table 2 shows that the node predictability values ranged from 52.2 to 70.7%. The average predictability value of 62.5% showed that neighboring nodes can explain 62.5% of the variance in the nodes in the symptom network on average. Sadness, feeling nervous, and panic had the highest predictability (*r*_*p*_), showing that 70.7, 69.8, and 68.2% of their variance can be explained by their neighbors.

### Accuracy and stability

**Figure 3:**
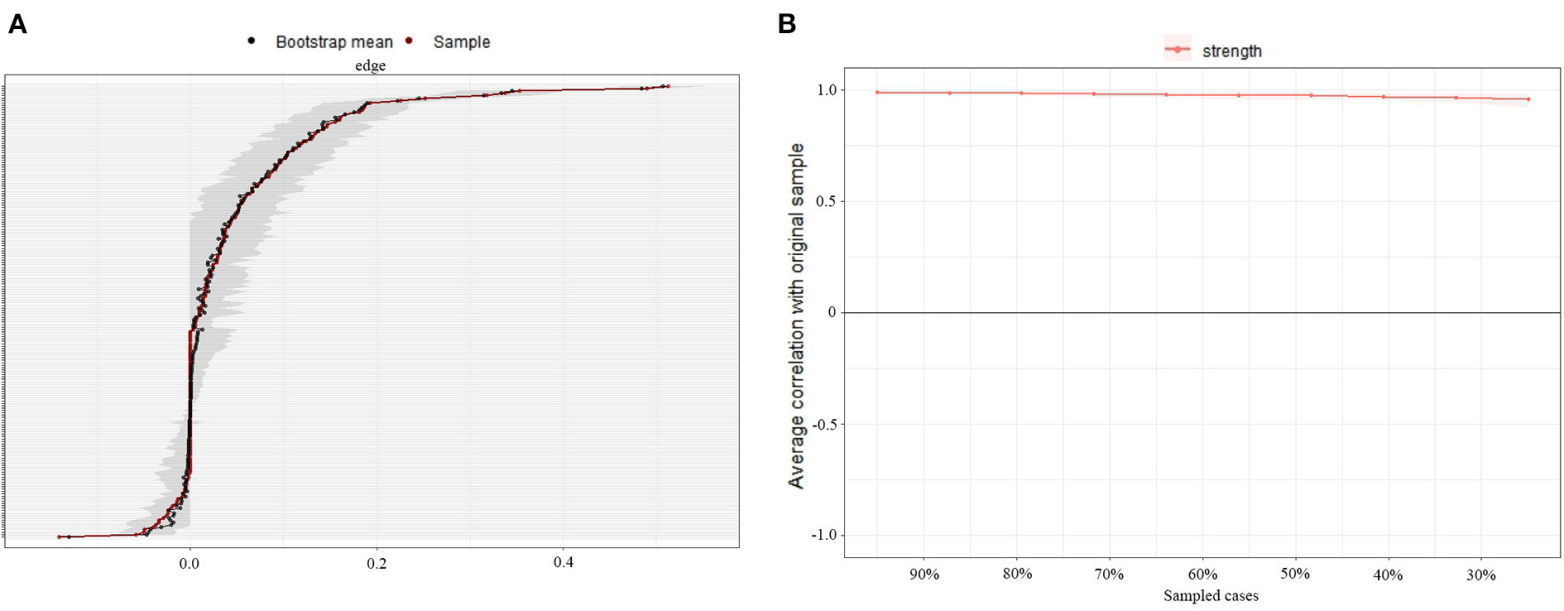
Accuracy and stability analysis of the symptom network. **(A)** Accuracy analysis of the edge weights; **(B)** Stability analysis of the strength centrality.

Figure 3A shows the bootstrap analysis results of the edge weights. The bootstrapped CIs were small, which showed good accuracy of the network. For the subset bootstrap (Figure 3B), the correlation stability coefficient (*r*_*cS*_) was >0.5, suggesting that the network remained stable.

### Difference tests

**Figure 4:**
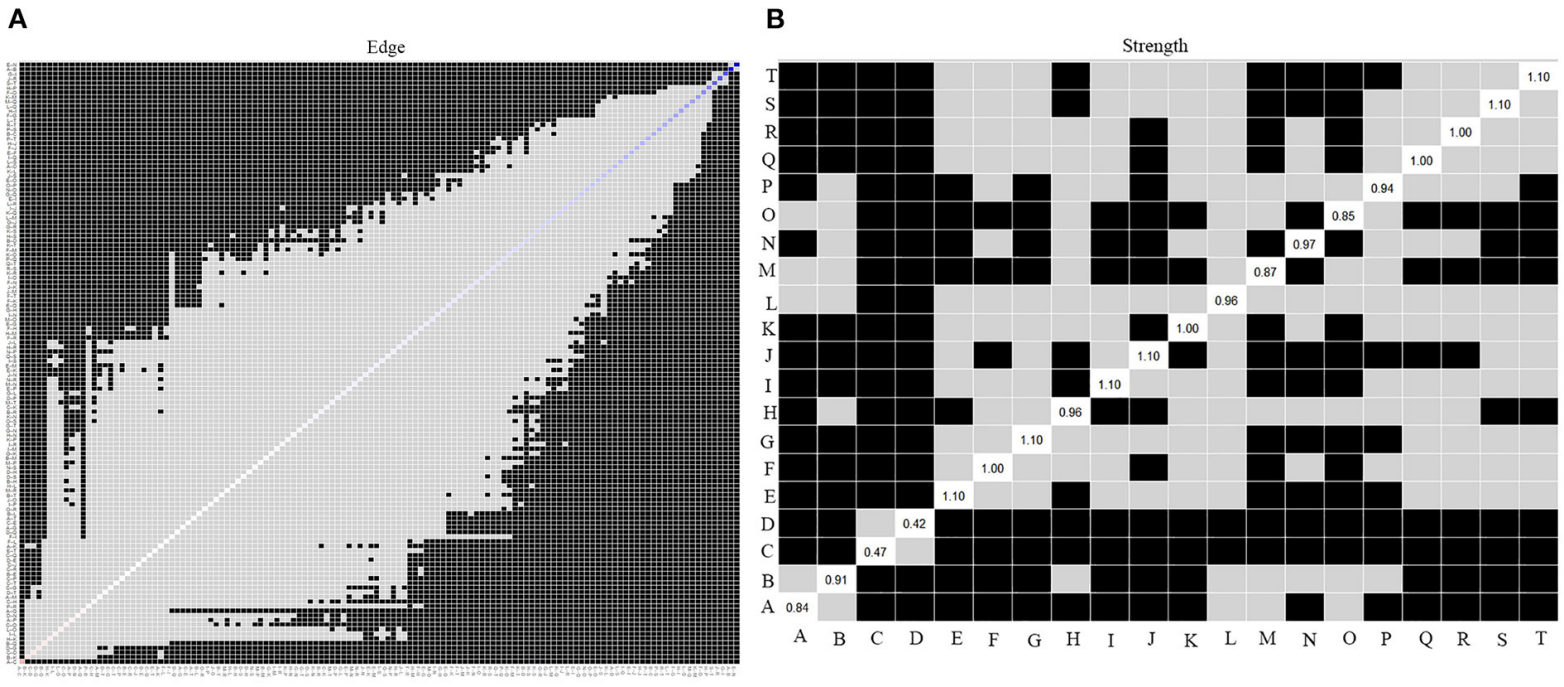
Bootstrapped difference test of the symptom network. **(A)** Bootstrapped difference test of the edge weights; **(B)** Bootstrapped difference test of the strength centrality. A, age; B, marital status; C, primary caregiver; D, years since HIV diagnosis; E, feeling nervous; F, feeling down; G, fear; H, anger; I, panic; J, sadness; K, loneliness; L, shame; M, feeling overwhelming pressure; N, uncontrollable worry; O, little interest; P, impulsivity; Q, feeling unsafe; R, self-abasement; S, self-loathing; T, self-blame.

Figure 4 shows the results of the bootstrapped difference test. Black boxes represent statistically significant differences in two edge weights or two strength centralities of nodes (*P* < 0.05). Gray boxes represent differences that were not statistically significant (*P* > 0.05). The bootstrapped difference test for edge weights showed that the four strongest edge weights, uncontrollable worry and feeling nervous, fear and panic, sadness and self-abasement, and self-loathing and self-blame, were significantly different from ~95% of the other edge weights (Figure 4A). Bootstrapped difference tests for node strength show that in the current network, the strength centrality of sadness, panic, self-loathing, and feeling nervous are significantly different from that of other symptoms (Figure 4B).

## Discussion

This is the first study to develop a psychological symptom network based on a large sample of PLWH and identify the central and bridge psychological symptoms in PLWH. We found that sadness was the central symptom in PLWH and that three bridge symptoms existed among different clusters of psychological symptoms in PLWH. “Uncontrollable worry” and “feeling unsafe” connected the personality traits and emotions clusters, and “self-abasement” connected the cognitive processes and emotions clusters. Based on network theory, both central and bridge symptoms can represent promising intervention targets from different perspectives (13, 44–46). The network also showed four correlations between uncontrollable worry and feeling nervous, fear and panic, sadness and self-abasement, and self-loathing and self-blame that were significantly stronger than those of the other edges.

We found that sadness had the highest values of centrality and predictability of all the symptoms, indicating that sadness may be the central symptom in PLWH from a mechanism-based perspective. Previous studies reported that a central symptom could serve as the target in psychological interventions (44, 45). A central symptom may spread the intervention effects to the peripheral nodes of the central symptom, eventually leading to the remission or disappearance of the other symptoms (44). Targeting central symptoms in psychological intervention treatment can accelerate the deactivation of the symptom network and improve the precision and efficiency of the intervention (18, 20–23). Rodebaugh's study demonstrated that central symptoms can predict changes in other symptoms and can be good targets for treatment (45).

Sadness, as a highly self-focused negative emotion, is one of the emotional expressions of depressive states (47, 48). Most people with depression present with sadness at various stages of HIV survivorship (47). Daily stress, internalized stigma, perceived discrimination, and neurologic complications of ART were all risk factors and triggers for generating sadness. Sadness, as the central symptom, could activate other depressive symptoms and finally lead to the diagnostic criteria of depression.

In addition, previous studies have shown that sadness is closely related to physical, cognitive, and social factors and may aggravate other psychological symptoms. Previous studies found that sadness was associated with higher rates of mood reactivity, social impairment, physical complaints, and terminal insomnia (49, 50). Wu et al. concluded that there was a correlation between a greater level of sadness and more negative emotional tendencies based on a laboratory-based study (51). Rush et al. found that the presence of sadness was associated with a poor prognosis (52). Furthermore, long-term sadness has vital implications in terms of a high risk of suicide (53, 54). The relationship between sadness and the desire to commit suicide was noteworthy. It is accepted that PLWH may experience brief periods of sadness, which is regarded as a defense mechanism to protect them from stress and trauma (33). However, sadness may increase the connectivity of the symptom network, which is the prime property for detecting changes for prognosis (55). Healthcare providers should assess the duration of sadness and develop symptom networks for PLWH to evaluate connectivity by using mobile devices.

Fear and self-loathing also had high strength centrality values. The fear of PLWH involves “stigmatizing attitudes toward PLWH” and “fear of disclosure” (56). Previous studies have shown a positive correlation between fear of disclosure and reluctance to seek care, and addressing this fear can improve care-seeking behavior (56, 57). The factors that help PLWH overcome this fear include partner acceptance, peer, community and health professional support, and accurate knowledge of transmission risk (58). Self-loathing manifests through consistent negative thoughts. If self-loathing continues for too long, it can lead to more severe conditions, such as depression or anxiety (59). Fang et al. (60) conducted a repeated-measures survey and concluded that self-loathing may signal the personal depressive reactions of students to failure and unmet expectations of success.

Bridge symptoms were broadly defined as symptoms that connect different clusters of symptoms (13). Bridge symptoms help identify the interaction among different symptom clusters and, therefore, represent potential targets of effective intervention (46). Our network identified three bridge symptoms. “Uncontrollable worry” and “feeling unsafe” connected the personality traits and emotions clusters, and “self-abasement” connected the cognitive processes and emotions clusters. In clinical interventions and treatments, bridge symptoms could be considered psychotherapeutic targets for the deactivation of symptom cluster interactions (46). Kaiser et al. (61) study showed that interventions targeting bridge symptoms were more likely to be effective for all symptom clusters. Jones et al. (18) study found that deactivated symptoms based on bridge strength rather than symptom strength alone were more effective in preventing symptom activation from spreading. These studies suggested that interventions focusing on bridge symptoms were more effective than those focusing on random targets or non-targeted psychotherapeutic interventions. Therefore, based on our findings, the possibility of using bridge symptoms as targets to intervene in the severity and prevalence of symptom clusters of PLWH is proposed.

We also found that self-abasement, as a crucial self-focused emotion, demonstrated a strong correlation with sadness. A previous study showed that self-abasement manifested as a symptomatological pattern of depression in a society with a conservative culture (62). In our study, traditional Chinese health beliefs continued to exert an influence on the perception of HIV/AIDS. The stigma and discrimination experienced by PLWH may lead to a low level of self-esteem. To protect themselves from being discriminated against, PLWH may tend to solve various types of medical and health issues on their own, which may lead to a chronic habit of placing more focus on themselves. Stroumpouki et al. (2) also found that PLWH excessively focused on themselves in emotional coping processes. It is crucial to relieve self-focused emotions, such as self-abasement, self-loathing, and self-discrimination, not by exaggerating PLWH self-emotional distress but by placing more emphasis on the consequences of disease for society overall (36, 63). Future efforts and interventions should increase PLWH's ability to view their HIV infection from both individual and social perspectives.

Previous studies have shown that uncontrollable worry increased the risk of anxiety disorders (64, 65). Additionally, uncontrollable worry can lead to impaired functioning in social, occupational and academic settings (66). Feeling unsafe may be related to social isolation, concerns about the economy, and fear of exposure (67, 68). Feeling unsafe was an important factor that was linked to the mental and physical health of PLWH, especially older PLWH (69). Previous studies have shown that feeling unsafe was associated with various mental health problems, including emotional problems and suicidal behavior (70). A study conducted in PLWH in rural Zambia concluded that feeling unsafe was associated with medication non-adherence, a higher level of stress, and more barriers to pill taking (71).

PLWH may have multiple concurrent symptoms. However, there is a shortage of health care professionals to provide symptom management services for all symptoms simultaneously. By identifying central and bridge symptoms, we can find psychotherapeutic targets among all concurrent symptoms. This approach could also enhance the efficiency and precision of symptom management. In future clinical symptom assessments and interventions, it is important to focus on not only severity indicators but also strength centrality and bridge strength centrality. Symptom networks can visualize the connections among symptoms and symptom clusters. The centrality indicators were analyzed to reflect the symptom interactions in the real world and provide targets for precise interventions. Currently, there is a shortage of health care workers, and frontline health care workers do not have enough energy to provide personalized and precise symptom management services. If we can identify the central symptom and bridge symptoms of PLWH, the efficiency and precision of clinical symptom intervention will be improved. Cognitive-behavioral therapy, such as cognitive reconstruction and emotional support, can be used in later interventions to target sadness, feeling unsafe, self-abasement, and uncontrollable worry (72, 73). By locating key populations through central symptoms, we can find the critical points in the symptoms for targeted interventions. In clinical practice, regular dynamic assessments and focused interventions are conducted in key populations.

### Limitations and further study

This is the first study to explore the relationships among the psychological symptom networks of PLWH in a large sample, however it has some limitations. First, the data were cross-sectional. Therefore, we can only describe the relationship among symptoms without making any causal inferences. Future studies are warranted to validate our results in a longitudinal sample. Second, our study used one item to assess each symptom. Future research should validate our results using multi-item measures. Third, PLWH who were willing to participate in the study might differ from those who were not, which could underestimate the centrality of central symptom. Due to the budget and scope of the study, our study only covered eastern, middle, and southern China. Further research could expand to the whole Chinese population. We did not conduct a subgroup analysis based on the regions in this study. Further research could compare multiple centers and the economic and cultural impacts of different regions.

## Conclusion

Our study identified central and bridge psychological symptoms in a large sample of PLWH. We found that sadness was the central psychological symptom in PLWH. Feeling unsafe, self-abasement, and uncontrollable worry were the bridge symptoms of the 16 psychological symptoms. All central and bridge symptoms could be promising therapeutic targets, as they may accelerate the deactivation of network interactions among psychological symptoms. Healthcare providers should assess the duration of sadness and develop symptom networks for PLWH to evaluate symptom connectivity. Future interventions should also relieve self-focused emotions not by exaggerating PLWH self-emotional distress but by increasing PLWH's ability to view their HIV infection from both individual and social perspectives.

## Data availability statement

The datasets presented in this article are not readily available because to protect the privacy of PLWH. Requests to access the datasets should be directed to ZZ, zhengzhu@fudan.edu.cn.

## Ethics statement

The studies involving human participants were reviewed and approved by Fudan University School of Nursing approved this study (IRB# TYSA2016-3-1, IRB# TYSQ2020-4-06). The patients/participants provided their written informed consent to participate in this study.

## Author contributions

HW: conceptualization, data collection, data analysis, methodology, and writing the original draft. ZZ: methodology and writing—review and editing. TH, CL, TJ, LL, LZ, and YF: data collection. SH: writing—review and editing. YH and BW: supervision, conceptualization, and writing—review and editing. All authors contributed to the article and approved the submitted version.
